# Microbiota-derived aromatic amino acid decarboxylases: linking microbial fitness and host neurochemical communication

**DOI:** 10.1128/mbio.02052-25

**Published:** 2025-10-02

**Authors:** Samane Rahmdel, Arif Luqman, Friedrich Götz

**Affiliations:** 1 Microbial Genetics, Interfaculty Institute of Microbiology and Infection Medicine Tübingen (IMIT), University of Tübingen 9188, Tübingen, Germany; 2 Department of Biology, Institut Teknologi Sepuluh Nopember 106242, Surabaya, Indonesia; 3 Excellence Cluster 2124 'Controlling Microbes to Fight Infections' (CMFI), University of Tübingen 9188, Tübingen, Germany; The Ohio State University, Columbus, Ohio, USA

**Keywords:** aromatic L-amino acid decarboxylases, neurochemical, microbiota

## Abstract

The human microbiota produces a diverse array of bioactive molecules, including classic neurotransmitters (dopamine and serotonin) and trace amines (tryptamine, tyramine, and phenylethylamine). Although long considered products of host metabolism, these aromatic monoamines are now also known to originate in part from the microbiota, where they are synthesized by bacterial aromatic L-amino acid decarboxylases (AADCs). This review explores the distribution, biochemical diversity, and host interactions of microbiota-encoded AADCs, highlighting their roles in gut and skin ecosystems. Bacterial AADCs vary in gene organization, substrate range, and expression patterns across taxa like *Ruminococcus gnavus*, *Clostridium sporogenes*, *Enterococcus* spp., and *Staphylococcus* spp. These enzymes contribute to microbial fitness through acid stress resistance, energy generation via proton motive force, epithelial adherence and internalization, and niche dominance. Critically, their products modulate host physiology via trace amine-associated receptors (TAARs) and other signaling pathways, influencing neurotransmission, immune response, barrier integrity, and metabolism. Microbiota-derived monoamines can enter systemic circulation and cross the blood–brain barrier, implicating them in disorders ranging from irritable bowel syndrome to neurodegeneration. Emerging data also reveal their impact on wound healing and drug efficacy, notably in Parkinson’s disease. By positioning microbial AADCs as key players in host-microbe chemical communication, this review underscores their relevance for health and disease and highlights them as potential therapeutic targets.

## INTRODUCTION

**Fig 1:**
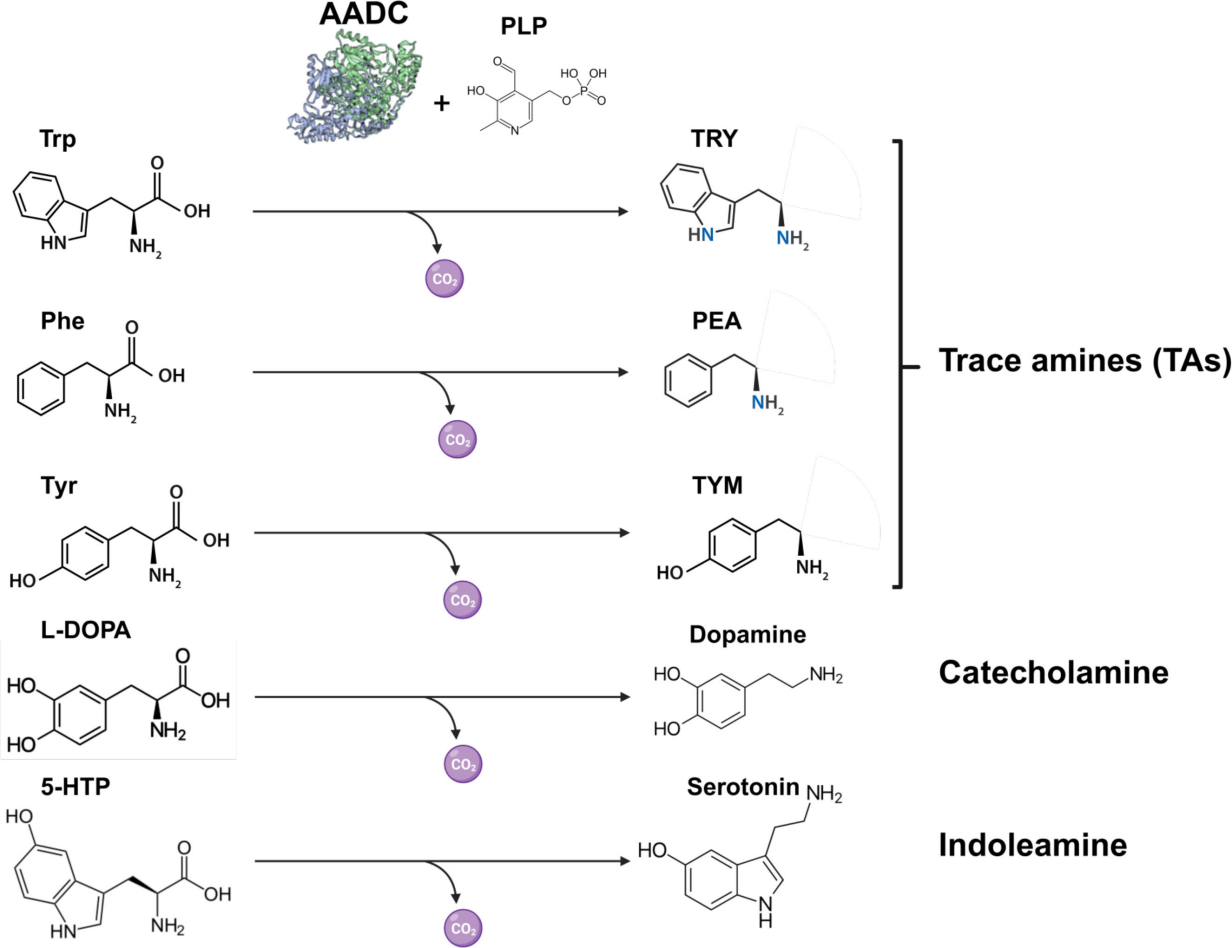
Structures of aromatic monoamines produced by bacterial aromatic L-amino acid decarboxylases (AADCs). These homodimeric, pyridoxal phosphate (PLP)-dependent enzymes differ in size and substrate specificity, with some producing two monoamines and others all five. All depicted monoamines function as neurotransmitters. The TAs interact with trace amine-associated receptors (TAAR1–TAAR9), modulating neuronal activity, olfaction, immune cell functions, and potentially contributing to neurological disorders. Dopamine activates dopamine receptors (DRs), regulating cognitive and physiological processes and mediating reward and pleasure. Serotonin acts through seven hydroxytryptamine receptor families (5-HT1–5-HT7), affecting mood, sleep, appetite, digestion, and cardiovascular function. Both TAs and dopamine can antagonize epinephrine binding to β2-adrenergic receptors, thereby promoting wound healing. The potential overproduction of these monoamines by bacterial AADCs in the skin or gut, and its consequences, remains poorly understood. The AADC structure was derived from the SadA model generated with AlphaFold3. Trp, tryptophan; TRY, L-tryptamine ; Phe, L-phenylalanine; Tyr, tyrosine; 5-HTP, 5-hydroxytryptophan; TRY, tryptamine; PEA, phenylethylamine; and TYM, tyramine.

The human microbiota is increasingly recognized as a source of bioactive compounds that influence different aspects of host physiology. Among these molecules are aromatic monoamines, including dopamine (DOPA), serotonin (SER), epinephrine, norepinephrine, and a group of less-studied compounds called trace amines (TAs) (1). Although aromatic monoamines have traditionally been regarded as products of host metabolism, growing evidence indicates that commensal bacteria also synthesize these neuroactive compounds (2). In particular, bacterial aromatic L-amino acid decarboxylases (AADCs) convert the aromatic amino acids tryptophan (L-Trp), tyrosine (L-Tyr), and phenylalanine (L-Phe) into trace amines (TAs), such as tryptamine (TRY), tyramine (TYM), and phenylethylamine (PEA) (Fig. 1) (3, 4). These microbially derived amines can influence host physiology through trace amine-associated receptors (TAARs), which respond to TAs at nanomolar concentrations—far more sensitively than to classical neurotransmitters. This heightened responsiveness suggests that microbiota-derived TAs may function not only as modulators but also as primary signals in host neurochemical pathways (5, 6).

Despite increasing interest in microbial neurochemistry, the origins, distribution, and physiological functions of microbiota-encoded AADCs remain poorly characterized. Although host AADCs are well defined in terms of structure, regulation, and tissue localization (6), their microbial counterparts show remarkable diversity in gene organization, substrate range, and regulatory mechanisms (2). Bacterial AADCs are found in a wide range of human-associated taxa, including gut commensals like *Ruminococcus gnavus* (*R. gnavus*) and *Clostridium sporogenes* (*C. sporogenes*) (7), *Enterococcus* sp (8, 9), and *Staphylococcus* species present in both the gut and skin microbiota (2, 4, 10), highlighting their ecological versatility. These enzymes have been linked to diverse microbial functions, from acid stress resistance (11–15) to host colonization (12, 16, 17), and are increasingly recognized as modulators of host barrier integrity (16, 18–21), metabolic function (22, 23), inflammation (24), immune signaling (25), skin wound healing (10), and even neurophysiology.

This review focuses on microbiota-derived AADCs, outlining their biochemical features, distribution, and microbial roles. We highlight emerging links to host processes such as neurotransmission, immunity, metabolism, and neurodegeneration, underscoring their potential as key players in host–microbe communication and as targets for therapeutic intervention.

## MICROBIOTA AADCs

Bacterial AADCs have been identified across diverse environments, from marine isolates (26, 27) and rhizosphere microbes (28) to soil-dwelling *Bacillus* species (29) and human-associated microbiota. Although all known bacterial AADCs are pyridoxal-5'-phosphate (PLP)-dependent homodimeric enzymes (1), they vary significantly in amino acid sequence length, phylogenetic relationships, substrate specificity, and enzymatic kinetics (2). Within the spectrum of AADCs produced by the human microbiota, gut-derived enzymes have been the most extensively studied.

Tyrosine decarboxylase (TDC), in particular, is widely distributed among enterococci and other lactic acid bacteria (LABs) (11, 13, 30–36). TDC catalyzes the decarboxylation of L-Tyr, L-DOPA, and to a lesser extent L-Phe, resulting in the formation of TYM, DOPA, and PEA, respectively. The TDC enzymes comprise approximately 620 amino acids per subunit, with a molecular weight of 70–75 kDa. They are encoded in a conserved operon structure usually composed of four genes: *tyrS* (tyrosyl-tRNA synthetase), *tdc* (tyrosine decarboxylase), *tyrP* (tyrosine-tyramine permease), and *nhaC* (Na^+^/H^+^ antiporter). Within the *tdc* operon, *tyrS* is thought to regulate decarboxylase activity in response to tyrosine levels and extracellular pH (32, 37). *tyrP* encodes a permease that exchanges tyrosine and tyramine, linking uptake with export (38). Although the precise function of *nhaC* remains uncertain, it may support pH homeostasis by mediating Na^+^/H^+^ exchange (14). Notably, some *tdc* operons, such as those in *E. faecalis* JH2-2 (39), *E. mundtii* (33), and *L. lactis* IPLA 655 (32), lack *nhaC*, pointing to operon variability and potential species-specific regulatory adaptations.

Another gut-associated AADC is tryptophan decarboxylase (TrpDC) found in gut microbes, *R. gnavus* and *C. sporogenes*. These enzymes share a similar product profile, producing TRY as the main product, along with PEA and TYM. Despite originating from different species, the enzymes are comparable in size, approximately 54 kDa (490 aa) in *R. gnavus* and 53 kDa (417 aa) in *C. sporogenes*, and are each encoded by a single gene, rather than a multi-gene operon as seen with *tdc* (7). In contrast, a TrpDC from the food isolate *Latilactobacillus curvatus* FAM25164 displays high substrate specificity, producing only tryptamine, and exhibits the highest sequence identity and coverage to TDCs among characterized AADCs, with a comparable length (624 aa) (40).

AADC homologs were also identified by our group in gut-associated *Staphylococcus* species, leading to the designation of the enzyme as staphylococcal aromatic amino acid decarboxylase (SadA). In staphylococci, *sadA* is chromosomally encoded without flanking mobile elements or a conserved insertion site, suggesting that it is not horizontally acquired. SadA is homologous to the TrpDC of *R. gnavus* and catalyzes the production of all three TAs (TRY, PEA, and TYM), as well as SER and DOPA. This capability is found across various *Staphylococcus* species, but TA production differs markedly among strains, with the highest levels observed in the *S. intermedius* group, including animal pathogens, *S. intermedius*, *S. pseudintermedius*, *S. delphini*, *S. schleiferi*, and *S. lutrae* (4). As *Staphylococcus* is one of the dominant genera on human skin, it was not unexpected to find SadA-harboring *Staphylococcus* species in the skin microbiota as well. Notably, the *sadA* gene is also widely present in human skin commensals such as *S. epidermidis*, *S. capitis*, and *S. hominis* (2, 10), although typically expressed at lower levels than in the *S. intermedius* group. The functional role of SadA in these remains under active investigation, with emerging evidence suggesting roles in host–microbe interactions, including the wound-healing phenotype observed in *S. epidermidis* (10, 41). Building on this, our group performed a metagenomic analysis of skin-associated microbial communities, which revealed SadA homologs across at least seven bacterial phyla, including *Bacillota*, *Actinobacteria*, *Proteobacteria*, *Bacteroidetes*, and *Acidobacteria*. Within *Bacillota*, homologs were identified in 23 genera, encompassing species from families such as *Clostridiaceae*, *Enterococcaceae*, *Lactobacillaceae*, and *Ruminococcaceae*, organisms commonly found not only on the skin but also as core members of the human gut microbiota (42). Interestingly, although SadA was commonly found among skin-associated *Staphylococcus*, a few commensal strains also carried a plasmid-encoded TDC operon, suggesting horizontal gene transfer, possibly from tyramine-producing LABs (2). This further underscores the metabolic plasticity of skin microbes and the potential for shared amine biosynthesis traits across ecological niches. The prevalence of AADCs in skin bacteria is ecologically consistent with the biochemical landscape of the skin. The skin surface is enriched in free aromatic amino acids, primarily originating from sweat and the ongoing proteolysis of epidermal proteins (10, 43–45). This abundance of substrates likely favors and sustains the activity of microbial AADCs such as SadA, positioning them as key enzymes in the utilization of host-derived nutrients and the production of compounds that shape the local microenvironment.

## CONTRIBUTION OF THE MICROBIOTA TO HOST AROMATIC MONOAMINE LEVELS

High levels of aromatic monoamines have been detected in stool and skin samples from healthy individuals (10, 17, 27). However, since humans also endogenously produce these compounds through host AADCs (6), it remains challenging to determine the extent to which the microbiota contributes to the overall monoamine pool. Evidence from animal studies, however, supports a significant microbial contribution, particularly to TA levels. In a study by Chen et al. (46), germ-free (GF) mice monocolonized with a PEA-producing *Morganella morganii* strain did not show elevated PEA in colon, serum, or brain unless treated with monoamine oxidase inhibitors (MAOIs), which block host enzymatic degradation of monoamines. Under MAOI treatment, PEA accumulated to toxic levels, leading to high mortality. In contrast, mice colonized with a non-producing *Bacteroides thetaiotaomicron* strain remained unaffected. These findings show that microbiota-derived monoamines can enter systemic circulation and, in the case of PEA, cross the blood–brain barrier (BBB) to reach the brain and exert neuroactive effects. Similar trends were observed by Sridharan et al. (47), who reported significantly lower TA levels in the cecal contents of GF mice compared with conventionally raised ones, and by Flores et al. (16), who showed that *R. gnavus* colonization elevated fecal tryptamine and tyramine levels. Although most research has focused on the gut microbiota, the contribution of skin microbes to monoamine production remains largely overlooked. However, monoamines generated at barrier sites like the skin can diffuse into circulation and act systemically. Given the passive permeability of TAs (6) and the widespread presence of TAARs throughout the body (48), the skin microbiota may represent a significant, although still underexplored, source of host monoamines.

## WHY BACTERIA PRODUCE MONOAMINES: FUNCTIONAL AND ECOLOGICAL INSIGHTS

**Fig 2:**
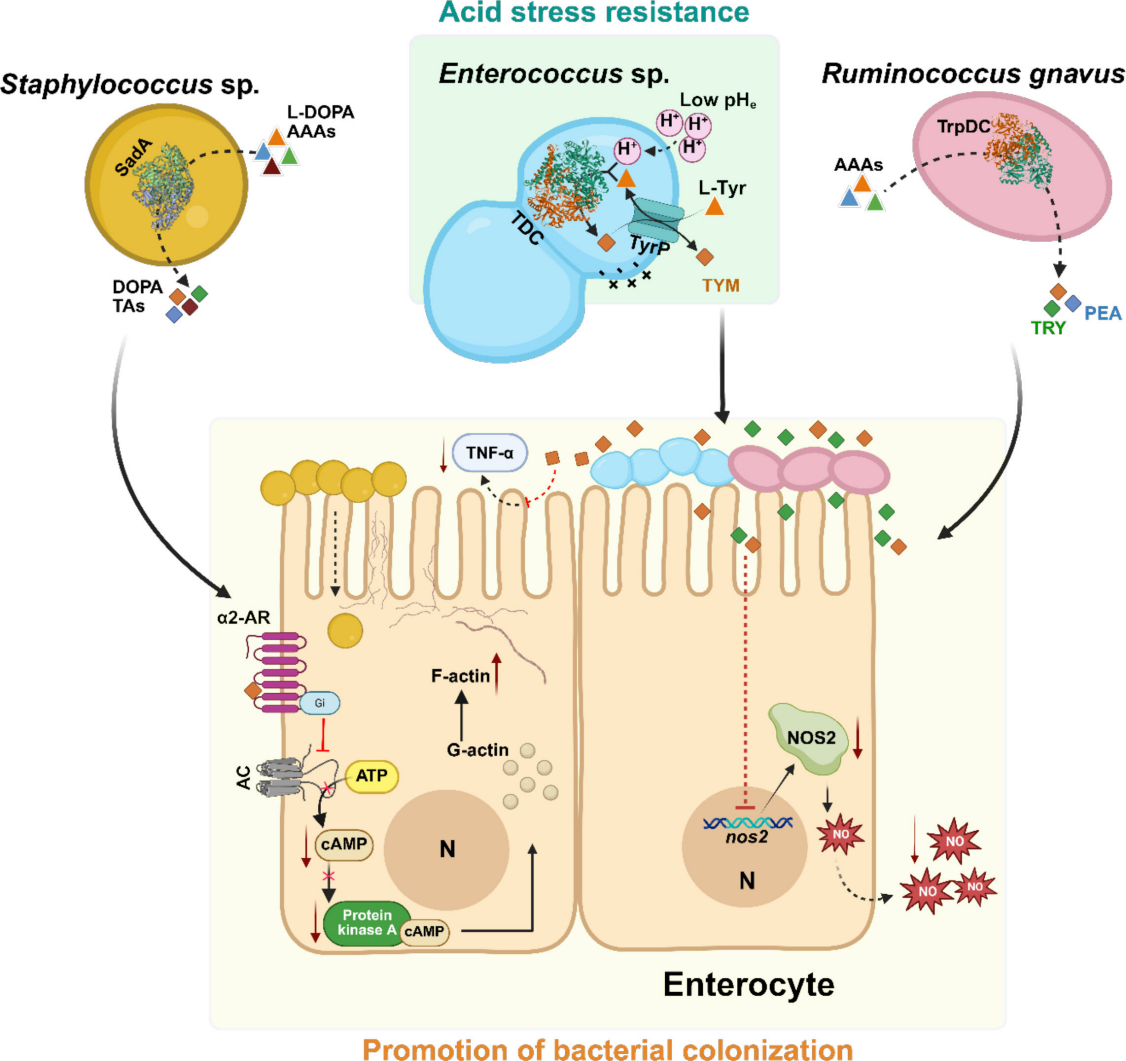
Functional and ecological insights of aromatic monoamines produced by human microbiota. Green panel: acid stress resistance: Tyramine (TYM) production by *Enterococcus* spp. is catalyzed by tyrosine decarboxylase (TDC) under low extracellular pH (pHe) in the presence of L-tyrosine (L-Tyr). The decarboxylation reaction consumes intracellular protons (H⁺), helping maintain cytosolic pH and promoting survival under acid stress, such as in the gastrointestinal tract. The export of TYM in exchange for L-tyrosine through tyrosine-tyramine permease (TyrP) also generates a proton motive force (PMF), visually indicated by the positive charge outside and negative charge inside the bacterial cell, which supports cellular energy generation; Yellow panel: Promotion of bacterial colonization: Trace amines (TAs) such as TYM, tryptamine (TRY), and phenethylamine (PEA) promote bacterial colonization by modulating host responses. TYM from *Enterococcus* enhances adhesion to enterocytes and suppresses TNF-α production. TYM and TRY produced by *Ruminococcus gnavus* via tryptophan decarboxylase (TrpDC) reduce nitric oxide synthase (NOS2) expression, lowering nitric oxide (NO) levels and weakening host defenses. In *Staphylococcus*, TAs synthesized by SadA activate host α2-adrenergic receptors (α2-AR), inhibiting cAMP signaling and promoting actin remodeling to facilitate bacterial adherence and internalization. AC, adenylate cyclase; Gi, inhibitory G protein; Gs, stimulatory G protein; and N, nucleus. Created in BioRender under license number BioRender.com/ernjp2d.

Although the full benefits of bacterial monoamine production are not yet fully understood, one well-supported advantage is acid stress resistance (Fig. 2). In TDC-harboring LABs, TYM production is induced under low pH in the presence of L-Tyr, consuming protons and helping maintain intracellular pH. This mechanism is particularly beneficial for survival in harsh acidic environments such as the gastrointestinal tract. Supporting this, TDC-deficient mutants show cytosolic acidification and reduced viability under acid stress, whereas wild-type strains retain near-neutral pH and remain viable (11–15). In addition to pH homeostasis, the pathway supports energy generation by creating a proton motive force (PMF) through tyrosine–tyramine exchange (14). The strength of this response varies by strain, reflecting differences in operon architecture and metabolic regulation (11). However, this acid resistance function is not conserved across all AADCs. In *R. gnavus* and *C. sporogenes*, tryptophan decarboxylase activity was not induced by low pH, and tryptamine production decreased under acid stress, suggesting it does not contribute to pH homeostasis in these strains (49). This may reflect a fundamental difference in gene organization, as TDC is typically part of a regulated operon, whereas TrpDC is a standalone gene with no apparent linkage to stress-response elements. Although direct evidence on SadA regulation is lacking, two plausible scenarios may account for its role in *Staphylococcus*. On one hand, to persist in low-pH environments such as human skin (pH 4.2-5.9) (50), staphylococci may employ mechanisms that upregulate AADC activity under acid stress, much like LABs utilize TDC. On the other hand, *Staphylococcus* species maintain a relatively neutral intracellular pH even under acidic conditions (50–52), unlike the marked pH drop observed in LABs (14, 53, 54). Since *sadA*, like *R. gnavus trpDC*, is encoded as a standalone gene, it is equally possible that its AADC activity functions independently of acid stress. Elucidating SadA’s physiological role will require further investigation.

On the other hand, *Staphylococcus* species maintain a relatively neutral intracellular pH even under acidic conditions (46–48), unlike the marked pH drop observed in LABs (14, 49, 50). Since *sadA*, like *R. gnavus TrpDC*, is encoded as a standalone gene, it is equally possible that its AADC activity functions independently of acid stress. Elucidating SadA’s physiological role will require further investigation.

Beyond potential roles in acid adaptation, SadA has been shown to enhance staphylococcal adherence and internalization into epithelial cells (Fig. 2). This occurs through the production of TAs, which activate host α_2_-adrenergic receptors (α_2_-AR) (41). Receptor activation triggers G_iα2_-mediated inhibition of adenylate cyclase, leading to reduced cAMP levels and downregulation of cAMP-dependent kinase activity. This promotes actin polymerization and the formation of pedestal-like structures on the host membrane, facilitating bacterial attachment and entry (17). A similar effect has been reported for TYM-producing *Enterococcus* strains, where activation of the biosynthetic pathway, rather than tyramine itself, enhanced adhesion to enterocytes and suppressed TNF-α production by them, thereby promoting bacterial colonization (12). In line with these findings, TYM and TRY produced by *R. gnavus* have been shown to promote its gut colonization by suppressing nitric oxide synthase 2 (NOS2), a host enzyme responsible for nitric oxide (NO) production. Reduced NOS2 expression leads to lower NO levels, weakening host defenses and allowing *R. gnavus* to expand in the gut (Fig. 2) (16). Moreover, TA production may help shape the microbial community within the niche by inhibiting biofilms and selectively eliminating competitors through curli suppression and reactive oxygen species (ROS) generation. This targeted interference allows producing strains to maintain dominance and influence local microbiota composition (49, 55, 56).

## INTERFACING WITH THE HOST: ROLES OF MICROBIAL AROMATIC MONOAMINES

**Fig 3:**
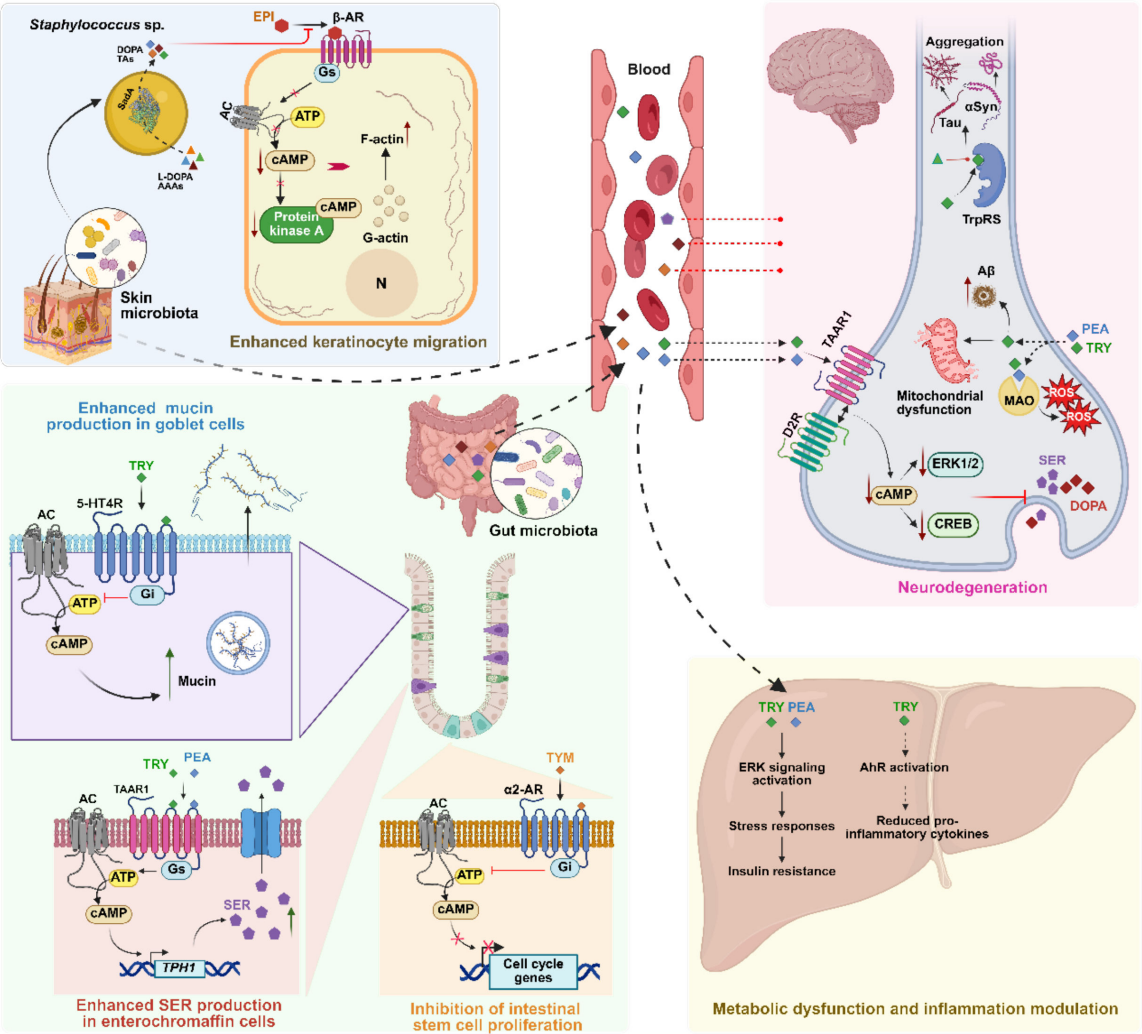
Microbiota-derived aromatic monoamines interfere with host physiology. Blue panel: Keratinocyte migration enhancement. Trace amines (TAs) and dopamine (DOPA) produced by skin commensals such as *Staphylococcus* enhance keratinocyte migration. They act as antagonists of β-adrenergic receptors (β-AR), countering epinephrine (EPI) signaling. This reduces intracellular cAMP, restores F-actin polymerization, and enhances keratinocyte migration, supporting wound healing. Green panel: intestinal functions. Gut-derived monoamines activate 5-hydroxytryptamine receptor 4 (5-HT4R) in goblet cells to promote mucin secretion. In enterochromaffin cells, they activate TA-associated receptor 1 (TAAR1), increasing serotonin (SER) via upregulation of tryptophan hydroxylase 1 (TPH1). In intestinal stem cells, tyramine (TYM) inhibits proliferation through α2-adrenergic receptor (α2-AR) signaling. Yellow panel: Liver metabolism and inflammation. Tryptamine (TRY) and phenethylamine (PEA) activate hepatic extracellular signal-regulated kinase (ERK) signaling, triggering stress responses and insulin resistance. TRY also activates aryl hydrocarbon receptor (AhR) signaling, reducing proinflammatory cytokines and promoting anti-inflammatory effects in hepatocytes and macrophages. Pink panel: Brain (neurodegeneration). Microbiota-derived tryptamine (TRY) and phenethylamine (PEA) cross the blood–brain barrier and modulate neural function. They promote the formation of TAAR1–dopamine receptor D2 (D2R) complexes, lowering cAMP and downregulating cAMP response element-binding protein (CREB) and ERK1/2 signaling, which impairs DOPA and SER release. TA degradation by monoamine oxidases (MAOs) produces reactive oxygen species (ROS), contributing to mitochondrial dysfunction and neuronal damage. TRY also competitively inhibits tryptophanyl-tRNA synthetase (TrpRS), favoring production of aggregation-prone, tryptophan-deficient proteins such as α-synuclein (αSyn) and tau. Additionally, TRY upregulates amyloid precursor-like protein 2 (Aplp2), contributing to amyloid beta (Aβ) formation. Together, these mechanisms link TA signaling to neurodegenerative processes involved in both Alzheimer’s and Parkinson’s diseases. AC, adenylate cyclase; Gi, inhibitory G protein; Gs, stimulatory G protein; and N, nucleus. Created in BioRender under license number BioRender.com/elggajs.

Microbiota-derived aromatic monoamines are emerging as key chemical signals in host interaction (Fig. 3). One well-characterized example is gut-derived TRY, which activates the SER receptor 5-HT4R on intestinal epithelial cells. Notably, *R. gnavus*, a commensal producer of TRY, has been shown to alleviate constipation and improve mucosal barrier function in mice, further supporting TRY’s physiological role (20). This signaling, triggered by *R. gnavus*-derived TRY, increases intracellular cAMP, which drives fluid secretion and accelerates intestinal transit. In an inflammatory bowel disease (IBD) mouse model, it also enhances barrier protection by promoting mucus release from goblet cells and reducing colitis severity (18, 57).

In contrast, a recent study demonstrated that *Enterococcus*-derived TYM exacerbates colitis by activating the α_2_-AR on intestinal stem cells, suppressing their proliferation by downregulating key cell-cycle genes. This *Enterococcus*-mediated effect impairs epithelial regeneration and weakens the intestinal barrier, allowing microbial translocation and amplifying inflammation (19). Consistently, TYM from *R. gnavus* was shown to impair gut barrier function more strongly and persistently than TRY (16), highlighting the context-dependent impact of monoamines on intestinal homeostasis. This complexity is further reflected in the elevated monoamine production observed in irritable bowel syndrome (IBS) patients, which may contribute to disease pathology. *R. gnavus*, enriched in IBS, converts dietary amino acids into PEA and TRY, which activate TAAR1, triggering serotonin overproduction and leading to diarrhea-like symptoms (58). TYM may also influence serotonin biosynthesis by increasing its release and upregulating tryptophan hydroxylase 1 (TPH1), the rate-limiting enzyme in serotonin production, in both colonic cells and germ-free mice (59).

TRY and PEA from *R. gnavus* have also been implicated in metabolic dysfunction (Fig. 3). These monoamines activate the extracellular signal-regulated kinase (ERK) pathway, which drives cellular stress responses, while inhibiting AKT (protein kinase B), a key regulator of insulin-stimulated glucose uptake, thereby resulting in insulin resistance in IBS and type 2 diabetes patients (23). Recent evidence implicates this signaling axis in metabolic dysfunction-associated steatotic liver disease (MASLD), where insulin resistance acts as a key initiating factor in hepatic steatosis (22). In contrast, tryptophan-derived metabolites, tryptamine and indole-3-acetate (I3A), have shown anti-inflammatory effects on macrophages and hepatocytes via activation of the aryl hydrocarbon receptor (AhR), a key regulator of inflammation and lipid metabolism (24), which may inhibit hepatocyte lipogenesis and protect against the progression of liver steatosis. These seemingly opposing effects likely reflect variations in host physiology, microbial strains, and metabolite profiles. Obese or insulin-resistant individuals may be more vulnerable to the harmful effects of monoamines due to inflammation and gut barrier dysfunction, whereas those with balanced immunity may benefit from AhR-mediated anti-inflammatory signaling. Strain-level genomic differences in TA-producing strains, along with diet-driven shifts in metabolite production, further influence whether pro- or anti-inflammatory pathways are activated (22).

In addition to epithelial and neuronal effects, microbial monoamines like PEA and TYM modulate immunity via TAAR1 and TAAR2. These receptors promote IgE class switching, IL-4 secretion, and leukocyte chemotaxis, suggesting a role in immune activation that may contribute to inflammation in Crohn’s disease and potentially IBS (25).

Although most research has focused on gut interactions, emerging evidence suggests that microbiota-derived monoamines may also influence host physiology at other barrier sites, particularly the skin. In GF mice colonized with a 50-species skin microbiota, TRY levels did not significantly increase and showed no direct benefit to skin barrier function. However, its downstream metabolite, indole-3-acetic acid (IAA), was enriched in colonized skin and was shown to enhance barrier integrity (21). Complementing these findings, our group demonstrated that TA-producing *S. epidermidis*, a common skin commensal, can accelerate wound healing. This effect is mediated by TAs (TYM, PEA, and TRY) and DOP, which act as partial antagonists of the β2-adrenergic receptor (β2-AR). By counteracting epinephrine-induced signaling, these compounds reduce intracellular cAMP levels and restore F-actin polymerization, thereby enhancing keratinocyte migration and promoting wound closure (Fig. 3) (10). These findings underscore the potential of commensal *Staphylococcus* species as active participants in skin repair and host modulation via TA production.

A largely overlooked aspect of microbiota-derived TAs is their potential contribution to neurodegenerative disorders. Most aromatic and biogenic amines, such as DOPA, SER, epinephrine, norepinephrine, octopamine, TYM, and histamine, are excluded from the central nervous system (CNS) due to poor lipid solubility and lack of blood-brain barrier (BBB) transport. However, PEA and TRY can cross (60). Although not exclusive to microbial origin, elevated levels of TRY have been shown to induce autophagic cell death in neuronal and glial cultures, characterized by mitochondrial swelling and autophagosome formation, features suggestive of a role in disorders such as Parkinson’s and Alzheimer’s diseases (PD and AD, respectively) (61). Furthermore, TAs, including microbiota-derived ones from gut and skin commensals, may exacerbate AD pathology through their interactions with TAARs (Fig. 3). Activation of TAAR1 by TAs facilitates its complex formation with dopamine receptor D2 (D2R), disrupting intracellular signaling by reducing cAMP levels. This downregulates pathways critical for neuronal function and survival, including cAMP response element-binding protein (CREB) and extracellular signal-regulated kinase (ERK1/2), while also decreasing the release of DOPA and SER, potentially impairing synaptic transmission.

Additionally, the degradation of TAs by monoamine oxidases (MAOs) generates hydrogen peroxide, contributing to oxidative stress. Normally, ROS are neutralized by antioxidants like reduced glutathione, but age-related declines in these defenses can lead to ROS buildup and neuronal damage, compounding TA-induced neurotoxicity. In AD, this effect is exacerbated by amyloid beta 42 (Aβ42)-mediated depletion of mitochondrial glutathione, promoting mitochondrial DNA damage and dysfunction. A study using *Saccharomyces cerevisiae* further supports this interaction: TYM and Aβ42 were synergistically toxic, inducing dose-dependent oxidative stress, mitochondrial dysfunction, and impaired mitophagy and mitochondrial biogenesis in yeast cells expressing Aβ42, but not in controls. These findings suggest that TYM may exacerbate Aβ42 toxicity through mechanisms independent of TAARs, reinforcing its potential role in AD pathology. Elevated iron and copper levels in AD brains may further intensify oxidative injury via Fenton chemistry, producing highly reactive hydroxyl radicals. Additionally, chronic MAO hyperactivation may impair lysosomal regeneration and autophagic clearance by inactivating transcription factor EB (TFEB), contributing to the accumulation of misfolded proteins and disturbances in lipid metabolism. TAs also influence molecular cascades linked to hallmark AD pathology: they can promote glycogen synthase kinase 3β (GSK3β) phosphorylation, driving tau aggregation, and activate the Akt-mTOR pathway, which suppresses autophagy and may lead to toxic protein aggregates. TAAR1 signaling has also been associated with disrupted glutamatergic transmission through N-methyl-D-aspartate receptors (NMDARs), further linking TAs to neurodegenerative mechanisms (62).

Another mechanism by which TRY may contribute to AD is through interference with protein synthesis and gene regulation. As a competitive inhibitor of tryptophanyl-tRNA synthetase (TrpRS), TRY reduces incorporation of tryptophan into proteins, favoring production of tryptophan-free proteins such as α-synuclein and tau, both prone to aggregation. TRY has been shown to induce neurofibrillary tangles, amyloidosis, and mitochondrial pathology in both neuronal and non-neuronal models (63, 64). Moreover, it upregulates multiple genes, including amyloid precursor-like protein 2 (*Aplp2*), a precursor of Aβ, linking TRY to amyloidogenic processes in AD (65).

## IMPACT OF MICROBIOTA-DERIVED AADCs IN PARKINSON’S DISEASE

Beyond their role in neurodegeneration, microbiota-derived AADCs can interfere with Parkinson’s disease (PD) therapy. Oral levodopa (L-DOPA) is decarboxylated to DOPA in the gut by both host and microbial AADCs before it reaches the brain. Because DOPA cannot cross the blood–brain barrier (BBB), this peripheral DOPA decreases drug efficacy. To counteract this, L-DOPA is co-administered with human AADC inhibitors such as carbidopa and benserazide, which have become standard components of anti-Parkinsonian therapy. By preventing peripheral L-DOPA decarboxylation, these inhibitors reduce systemic dopamine levels and thereby diminish side effects such as nausea and orthostatic hypotension (66).

Carbidopa irreversibly binds pyridoxal 5′-phosphate (PLP), inhibiting PLP-dependent AADCs (67), whereas benserazide acts as a competitive inhibitor of human AADCs (68, 69). Neither compound crosses the BBB, ensuring that central conversion of L-DOPA to dopamine remains unaffected. However, it has been found that neither carbidopa nor benserazide inhibit microbiota-derived AADCs (9); in fact, both have been reported to induce peripheral AADC activity (70).

Consequently, microbial AADC activity continues to reduce L-DOPA bioavailability. In addition, microbial dopamine and tyramine can slow gut motility, further impairing L-DOPA absorption. Prolonged use of L-DOPA with AADC inhibitors may even select for AADC-expressing bacteria in the small intestine, perpetuating a cycle of reduced efficacy and escalating dosage requirements (8, 9).

## CONCLUSION AND FUTURE QUESTIONS

Microbiota-derived aromatic monoamines, once overshadowed by their host-produced counterparts, are now recognized as powerful modulators of host physiology. Bacterial AADCs not only promote microbial survival and colonization but also shape host processes such as neurotransmission, epithelial barrier integrity, immunity, metabolism, and even neurodegeneration. Their effects are highly context-dependent—ranging from protective to pathogenic—highlighting the complexity of host–microbe interactions mediated by these bioactive amines. Notably, the ability of certain monoamines, including TRY and PEA, to cross the blood–brain barrier and influence neuronal signaling expands their physiological relevance, whereas the emerging interference of microbial AADCs with Parkinson’s disease therapy underscores their clinical significance.

Despite recent advances, much remains unknown about the regulation, ecological drivers, and full host impact of microbial AADCs. Key open questions for future research include the following. How is the expression of sadA and other orphan AADC genes controlled by environmental cues in specific niches? Do the benefits of TA production extend beyond acid resistance and adhesion to include inter-bacterial signaling or shaping the broader microbial community structure? Furthermore, can we develop selective inhibitors that target bacterial AADCs without affecting host enzymes, offering a novel strategy to modulate aromatic monoamine levels in metabolic, gastrointestinal, or neurological disorders?

As precision microbiome modulation tools advance, targeting bacterial AADC activity represents a promising frontier. Realizing this potential will require integrated approaches that combine microbial genetics, host signaling, and systems biology to chart the complex landscape of microbial neurochemistry.
